# Individual effects of *GSTM1* and *GSTT1* polymorphisms on cervical or ovarian cancer risk: An updated meta-analysis

**DOI:** 10.3389/fgene.2022.1074570

**Published:** 2023-01-12

**Authors:** Jing Ye, Yi-Yang Mu, Jiong Wang, Xiao-Feng He

**Affiliations:** ^1^ The First People's Hospital of Bijie, Bijie, Guizhou, China; ^2^ Orthopedics, Heping Hospital Affiliated to Changzhi Medical College, Changzhi, Shanxi, China; ^3^ Department of Gynecology, Heji Hospital Affiliated to Changzhi Medical College, Changzhi, Shanxi; ^4^ Institute of Evidence-based medicine, Heping Hospital Affiliated to Changzhi Medical College, Changzhi, Shanxi

**Keywords:** *GSTT1*, *GSTM1*, cervical cancer, ovarian cancer, BFDP, FPRP

## Abstract

**Background:** Studies have shown that glutathione S-transferase M1 (*GSTM1*) and. glutathione S-transferase T1 *(GSTT1)* null genotype may increase the risk of cervical cancer (CC) or ovarian cancer (OC), however, the results of published original studies and meta-analyses are inconsistent.

**Objectives:** To investigate the association between *GSTM1* present/null and *GSTT1* present/null polymorphisms, with the risk of cervical cancer or ovarian cancer.

**Methods:** The odds ratios (ORs) and 95% confidence intervals (CIs) were used to assess the association between *GSTM1* present/null and *GSTT1* present/null polymorphisms and the risk of cervical cancer or ovarian cancer. To assess the confidence of statistically significant associations, we applied false positive reporting probability (FPRP) and bayesian false discovery probability (BFDP) tests.

**Results:** Overall analysis showed that *GSTM1* null was associated with an increased risk of cervical cancer, and subgroup analysis showed a significant increase in cervical cancer risk in Indian and Chinese populations; *GSTT1* was not found null genotype are significantly associated with cervical cancer. Overall analysis showed that *GSTM1* and *GSTT1* null were not associated with the risk of ovarian cancer, subgroup analysis showed that *GSTM1* null was associated with an increased risk of OC in East Asia, and *GSTT1* null was associated with an increased risk of OC in South America. However, when we used false positive reporting probability and bayesian false discovery probability to verify the confidence of a significant association, all positive results showed “low confidence” (FPRP > .2, BFDP > .8).

**Conclusion:** Overall, this study strongly suggests that all positive results should be interpreted with caution and are likely a result of missing plausibility rather than a true association.

## Introduction

Gynecological cancers have different degrees of negative impact on women’s health around the world. Among them, with CC the highest incidence and OC with the highest mortality have attracted much attention. According to the 2020 global cancer incidence and mortality statistics released by the World Health Organization, about 604,000 women were diagnosed with CC, and about 342,000 women died of CC, witch has become the most common cancer in 23 countries and 36. The number one cause of cancer death in 100 countries. According to the data survey released by the national cancer center of my country, in recent years, the incidence of CC has increased at an average annual rate of 8.7% (Zhao and Song, 2021). According to global statistics in 2020, about 310,000 women were diagnosed with OC, and about 210,000 women died of OC. The analysis of the incidence and death data of OC in the “China cancer registry annual report” shows that from 2005 to 2016, OC in China incidence and mortality are rapidly increasing, and most OC occur in people over the age of 50 (Huang et al., 2022). Although the main pathogenic factors of the two cancers are different, epidemiological studies have shown that the occurrence of both cancers is related to individual genetic susceptibility, and studies have shown that the genetic polymorphism of cancer susceptibility genes is associated with high cancer risk. There may be associations; therefore, finding true gene associations will help people to further understand the pathogenesis of CC and OC, and actively exploring the multi-pathway pathogenesis of CC and OC is of great significance for cancer prevention, diagnosis, and treatment (Ueda et al., 2008).

Glutathione s-transferase system (*GSTs*: Glutathione s -transferases), as the first line of defense in cell protection, participates in the detoxification process of exogenous toxins *in vivo*, making reduced glutathione and electrophilic substances combine to convert toxic substances in the body into hydrophilic substances, which are excreted through urine or bile to complete the detoxification process (Board and Menon, 2013; Zou, 2013). Currently, eight glutathione s-transferases have been identified in mammals, including alpha, kappa, mu, omega, pi, sigma, theta, and zeta. Among them, mu (µ)-type *GSTM1* and theta (θ)-type *GSTT1* is the most studied genes in the relationship between gynecological tumors and glutathione transferase, *GSTM1* is located on chromosome 1 (1p13.3), *GSTT1* is located on chromosome 22 (p11.23), its function is to link various parent electrochemical compounds (such as drugs, environmental toxins, oxidation chain products, etc.) combine with glutathione to enter the next metabolic step, allowing the toxic substances to be easily excreted from the body. The *GST* gene has polymorphisms at multiple loci, among which *GSTM1* and *GSTT1* share a common zero allele. The most common mutation of these two genes is the whole null genotype, and the mutation of the gene will change the activation or inactivation of the corresponding enzyme. The ability to source substrates, thereby affecting the detoxification of carcinogens, exposing cells in the body to toxic substances, causing DNA damage, potentially increasing somatic mutations that increase an individual by 39%, risk of developing tumors (Abbas et al., 2018; Sharma et al., 2019). Therefore individuals with homozygous null genotype polymorphisms are considered potential risk factors for the development of various malignancies in humans. At present, the correlation of *GSTM1* and *GSTT1* present/null polymorphisms with CC and OC is still unclear. Therefore, studying the glutathione metabolic pathway involving glutathione-s-transferase may be useful for early warning and early warning of gynecological malignancies. Prevention as well as treatment options and prognosis for cancer patients are of great importance.

So far, there have been 31 articles (Warwick A. P et al., 1994; Warwick A et al., 1994; Chen et al., 1999; Kim et al., 2000; Sierra-Torres et al., 2003; Lee et al., 2004; Sharma et al., 2004; Niwa et al., 2005; Huang, 2006; Joseph et al., 2006; Sobti et al., 2006; Zhou et al., 2006; de Carvalho et al., 2008; Nishino et al., 2008; Singh et al., 2008; Song et al., 2008; Ueda et al., 2008; Liu et al., 2009; Settheetham-Ishida et al., 2009; Kiran et al., 2010; Palma et al., 2010; Ueda et al., 2010; Stosic et al., 2014; Hasan et al., 2015; Nunobiki et al., 2015; Sharma et al., 2015; Satinder et al., 2017; Tacca et al., 2018; Wang et al., 2018; Zhang et al., 2019; Wongpratate et al., 2020) on the individual and combined effects of *GSTM1* and/or *GSTT1* present/null polymorphisms and CC risk, and nine meta-analyses (Economopoulos et al., 2010a; Gao et al., 2011; Sui et al., 2011; Wang et al., 2011; Liu and Xu, 2012; Zhang et al., 2012; Zhen et al., 2013; Sun and Song, 2016; Tian et al., 2019) reporting *GSTM1* and/or *GSTT1* present/null polymorphisms associated with CC risk. 14 articles investigated the individual impact of *GSTM1* and/or *GSTT1* present/null polymorphisms and OC risk (Sarhanis et al., 1996; Esteller et al., 1997; Hengstler et al., 1998; Goodman et al., 2000; Baxter et al., 2001; Spurdle et al., 2001; Morari et al., 2006; Chunhua, 2008; Gates et al., 2008; Ueda et al., 2008; Khokhrin et al., 2012; Oliveira et al., 2012; Cai et al., 2016; Pljesa et al., 2017), and five meta analyses (Economopoulos et al., 2010b; Yin et al., 2013; Han et al., 2014; Jin and Hao, 2014; Xu et al., 2014) reported individual effects of *GSTM1* and/or *GSTT1* present/null polymorphisms and OC risk. However, the conclusions of all studies were inconsistent and even contradictory. Furthermore, no study has examined the correlation between the corresponding positive results. Correlations are assessed for reliability. Newer original studies have recently been published investigating these associations, and therefore, an updated meta-analysis should be performed to explore these questions. Two methods FPRP and BFDP tests were used to assess the confidence of these findings. We aim to provide a real link to these questions and to discuss the positive findings identified in terms of biological mechanisms involved in CC and OC.

## Material and methods

### Literature search strategy

This meta-analysis was conducted based on the priority reported entries of systematic reviews and meta-analyses (PRISMA). Pubmed, Embase, Scopus, Chinese biomedical medical databases (CBM), China national knowledge infrastructure (CNKI), and Wanfang databases and so on in both Chinese and English (up to 15 September 2021) were searched to identify eligible studies that analyzed the *GSTM1* present/null and *GSTT1* present/null, with CC and OC risk. The following keywords were used: (*GSTT1* OR glutathione s-transferase T1 OR *GSTM1* OR glutathione s-transferase M1) AND (polymorphism OR variant OR mutation) AND (ovarian cancer OR oophoroma OR carcinoma of ovary OR cervical cancer OR carcinoma of uterine cerxix OR cervical malignancy). The search strategy was designed to be sensitive and broad. We first carefully reviewed the title and abstract of the search results, and then downloaded full articles to identify possible articles. These were evaluated in detail to identify relevant articles. The reference lists of identified articles and reviews was also examined as appropriate. The corresponding author may be contacted by e-mail if only the abstract was available online or the data was incomplete.

### Literature inclusion and exclusion criteria

Inclusion criteria were as listed below: 1) articles on the *GSTM1* present/null and *GSTT1* present/null, with the risk of CC or OC. 2) The diagnostic criteria for CC and OC meet histological or pathological criteria. 3) case-control studies or cohort studies where the language of the literature is limited to Chinese or English. 4) sufficient genotype data to calculate ORs and 95% CIs. Exclusion criteria were as listed below: 1) no raw data. 2) no control. 3) review articles, case reports, editorials, or animal research. 4) duplicate and insufficient data.

### Extraction information

Two investigators independently extracted data using excel. Any disagreement was solved by iteration, discussion, and consensus. The details of the data extraction form included the following: first author, year of publication, country, geographical region, ethnicity, control source, control type, matching, adjusted OR, SNP, sample size, each locus, the number of genotypes, and the literature quality score. Of these, the literature quality score needs to be obtained by calculation.

### Quality score assessment

The quality of all studies was assessed independently by two researchers. We supplemented and improved the quality assessment criteria from relevant guidelines and previous meta-analysis, combined with NOS criteria (Aerssens et al., 2000; Moher et al., 2009; Thakkinstian et al., 2011), Supplementary Table S1 lists the quality assessment scales for studies of the association of CC or OC risk. Studies were considered to be of low quality if the quality score was less than 9, whereas in the Meta-analysis, scores ≥ 11 were considered to be of high quality, and studies with scores between 9 and 11 were considered to be of moderate quality. Supplementary Table S1 lists the scoring scale for assessing the quality of the literature with the following entries: 1) source of the experimental group; 2) source of the control group. 3) diagnostic criteria for patients with CC and OC. 4) inclusion criteria for the control group. 5) whether the experimental and control groups were matched. 6) genotype testing. 7) samples used to determine genotype. 8) assessment of the association between genotype and OC and CC. 9) size of sample size.

### Statistical analysis

We applied the crude ratio (OR) and its 95% confidence interval (CI) to assess the association effect of the *GSTM1* present/null and *GSTT1* present/null, with the risk of CC or OC. Q-tests were used to assess heterogeneity between selected studies and statistically, significant heterogeneity was considered if *p* < .10 and/or *I* ^*2*^ > 50%, using a random-effects model (Mantel and Haenszel, 1959), and if heterogeneity was not significant (*I* ^*2*^ ≤ 50%), a fixed-effects model (Der Simonian and Laird, 2015) was considered, followed by a search for sources of heterogeneity based on meta-regression analysis. Subgroup analyses were performed for HPV infection, smoking, geographic region, and ethnicity according to CC epidemiology, and for ethnicity and geographic region according to OC epidemiology. Two methods were used to conduct sensitivity analyses: one was to exclude one study at a time. The second was to conduct statistical analyses after excluding low-quality and small-sample studies. Publication bias was confirmed according to Begg’s funnel plot (Begg and Mazumdar, 1994) and Egger’s test (considered significant publication bias if *p* < .05) (Egger et al., 1997) and if publication bias was observed, non-parametric pruning and padding methods were applied to identify missing studies (Dual and Tweedie, 2000). To assess the confidence of statistically significant associations in the current and previous meta-analyses, we applied the FPRP (Wacholder et al., 2004) and the BFDP test (Ioannidis et al., 2008), and the FPRP was estimated using the excel spreadsheet appendix. All statistical analyses were calculated using Stata version 12.0 (STATA Corporation, college station, TX).

## Results

### Literature search results

**FIGURE 1:**
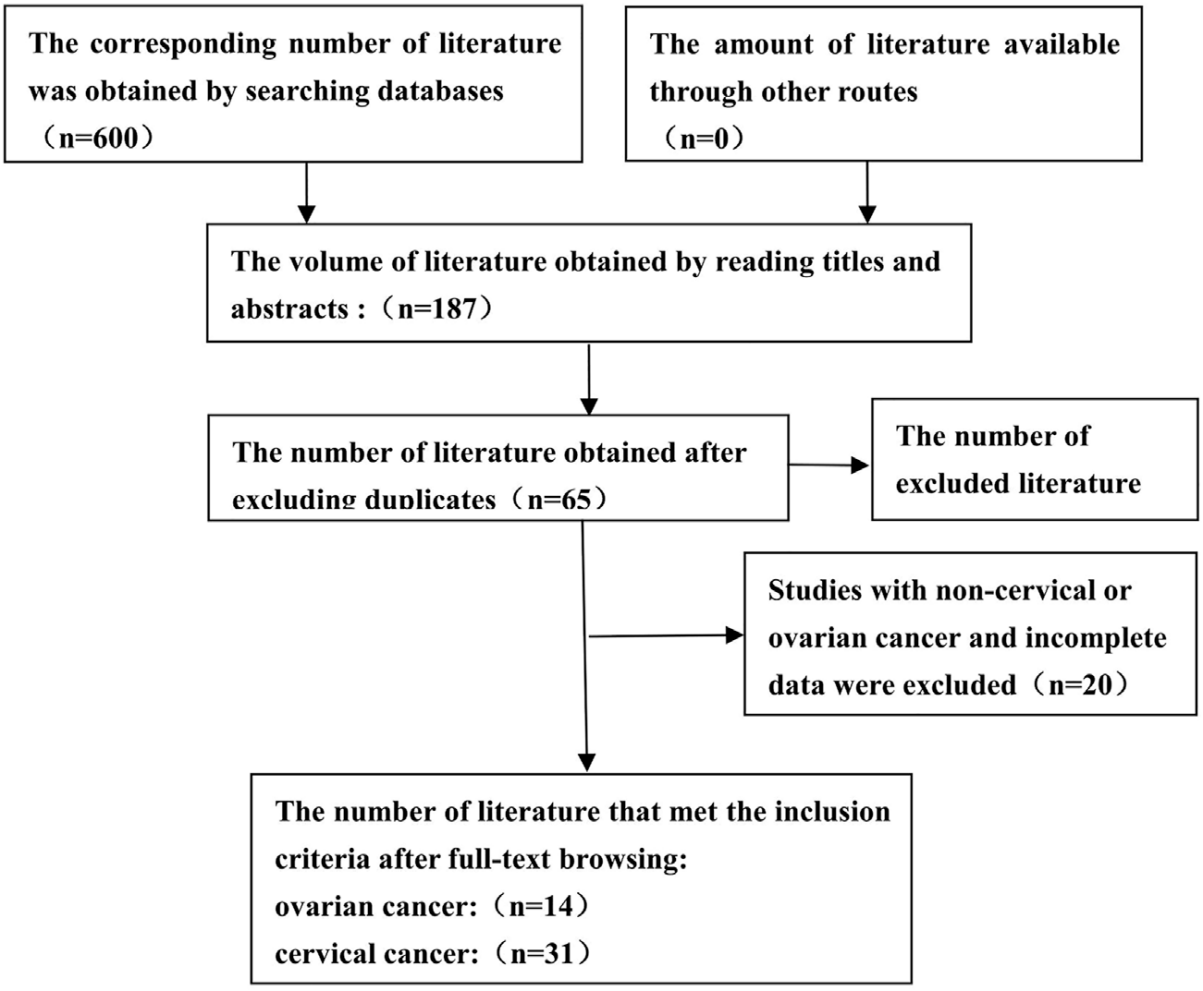
Flow diagram for identifying and including studies in the current meta-analysis.

A total of 600 articles were searched (Figure 1). After reading the topic, 413 articles inconsistent with this study (including other genotype studies, reviews, case reports, meta-analyses, and letters) were excluded, 122 duplicate articles were excluded after further reading of the title and abstract, and the remaining articles were read in full of the 66 articles, 22 studies for which complete data were not available were excluded, and the final 44 original articles were included in this study. 31 studies related to CC were included (including 30 for *GSTM1* and 22 for *GSTT1*) (Warwick A. P et al., 1994; Warwick A et al., 1994; Chen et al., 1999; Kim et al., 2000; Sierra-Torres et al., 2003; Lee et al., 2004; Sharma et al., 2004; Niwa et al., 2005; Huang, 2006; Joseph et al., 2006; Sobti et al., 2006; Zhou et al., 2006; de Carvalho et al., 2008; Nishino et al., 2008; Singh et al., 2008; Song et al., 2008; Ueda et al., 2008; Liu et al., 2009; Settheetham-Ishida et al., 2009; Kiran et al., 2010; Palma et al., 2010; Ueda et al., 2010; Stosic et al., 2014; Hasan et al., 2015; Nunobiki et al., 2015; Sharma et al., 2015; Satinder et al., 2017; Tacca et al., 2018; Wang et al., 2018; Zhang et al., 2019; Wongpratate et al., 2020), 14 studies related to OC (including 14 *GSTM1* and 11 *GSTT1*) (Sarhanis et al., 1996; Esteller et al., 1997; Hengstler et al., 1998; Goodman et al., 2000; Baxter et al., 2001; Spurdle et al., 2001; Morari et al., 2006; Chunhua, 2008; Gates et al., 2008; Ueda et al., 2008; Khokhrin et al., 2012; Oliveira et al., 2012; Cai et al., 2016; Pljesa et al., 2017). Table 1 shows the general characteristics of the studies included in this meta-analysis. Among the studies on CC risk, there were 30 articles on *GSTM1* present/null polymorphisms (including 3,484 cases and 4,208 controls, see Table 2), 22 articles on *GSTT1* present/null polymorphisms (including 2,500 cases), and 3,148 control cases, see Table 3). Among OC risk studies, there were 14 articles on *GSTM1* present/null polymorphisms (including 3,035 cases and 3,422 controls, see Table 2), 11 articles on *GSTT1* present/null polymorphisms (including 2,543 cases and 3,275 controls, see Table 3). Finally, according to the quality assessment of molecular association studies, among the studies on the association of *GSTM1* present/null polymorphisms with CC risk, there were 13 high-quality, 7 medium-quality, and 10 low-quality studies. Among studies on the association between polymorphisms and CC risk, there were 9 high-quality, 7 moderate-quality and 7 low-quality studies, Among the studies on the association between *GSTM1* present/null and OC risk, there were 6 high-quality studies. High-quality, 3 moderate-quality, and 5 low-quality studies, among the studies on the association of *GSTT1* present/null polymorphisms with OC risk, there were 5 high-quality, 2 moderate-quality, and 4 low-quality studies.

**TABLE 1:** General situation and quality evaluation of the included study.

First author/year	Country	Geographic region	Ethnicity	Tumor classification	Source of controls	Matching	Adjustments	SNP	Quality score
Warwick A. P. Warwick A. P et al. (1994)/1994	United Kingdom	Europe	Caucasian	CC	HB	NA	NA	*GSTM1*	7
Warwick A, Warwick A et al. (1994)/1994	United Kingdom	Europe	Caucasian	CC	HB	NA	NA	*GSTT1*	8
Chen C Chen et al. (1999)/1999	United States	North America	Caucasian	CC	PB	Age	Age	*GSTM1, T1*	15
Kim JW Kim et al. (2000)/2000	Korea	East Asia	Asian	CC	PB	Age	Age	*GSTM1, T1*	14
S-T CH Sierra-Torres et al. (2003)/2003	United States	North America	Caucasian	CC	PB	Age	Smoking	*GSTM1*	12
Lee SA Lee et al. (2004)/2004	India	South Asia	Indian	CC	PB	NA	NA	*GSTM1, T1*	10
Sharma A Sharma et al. (2004)/2004	Korea	East Asia	Asian	CC	HB	NA	NA	*GSTM1, T1*	8
Niwa Y Niwa et al. (2005)/2005	Japan	East Asia	Asian	CC	HB	NA	Age	*GSTM1, T1*	13
Zhou Q Zhou et al. (2006)/2006	India	South Asia	Indian	CC	HB	NA	NA	*GSTM1, T1*	9
Joseph T Joseph et al. (2006)/2006	China	East Asia	Asian	CC	HB	NA	Age	*GSTM1, T1*	11
Huang YK Huang (2006)/2006	China	East Asia	Asian	CC	HB	NA	NA	*GSTM1*	9
Sobti RC Sobti et al. (2006)/2006	India	South Asia	Indian	CC	PB	Age	NA	*GSTM1, T1*	16
Nishino K Nishino et al. (2008)/2008	Japan	East Asia	Asian	CC	PB	NA	Age	*GSTM1, T1*	11
De C CR de Carvalho et al. (2008)/2008	Brazil	South America	Mixed	CC	HB	NA	Age	*GSTM1, T1*	9
S-I W Settheetham-Ishida et al. (2009)/2009	Thailand	Southeast Asia	Asian	CC	PB	Age	Age	*GSTM1, T1*	14
Song GY Song et al. (2008)/2008	China	East Asia	Asian	CC	PB	NA	NA	*GSTM1*	12
Singh H Singh et al. (2008)/2008	India	South Asia	Indian	CC	PB	NA	NA	*GSTM1, T1*	11
Liu Y Liu et al., (2009)/2009	China	East Asia	Asian	CC	HB	NA	NA	*GSTM1*	4
Palma S Palma et al. (2010)/2010	Italy	Europe	Caucasian	CC	PB	Age	Age	*GSTM1, T1*	14
Ueda M Ueda et al. (2010)/2010	Japan	East Asia	Asian	CC	PB	NA	NA	*GSTM1, T1*	11
Kiran B Kiran et al. (2010)/2010	Turkey	West Asia	Caucasian	CC	HB	NA	NA	*GSTM1, T1*	10
Stosic I Stosic et al. (2014)/2014	Serbia	Europa	Serbian	CC	PB	NA	NA	*GSTM1, T1*	11
Natphopsuk S Nunobiki et al. (2015)/2015	Thailand	Southeast Asia	Asian	CC	HB	Age	Age	*GSTM1*	13
Hasan S Hasan et al. (2015)/2015	Pakistan	South Asia	Caucasian	CC	PB	NA	NA	*GSTM1, T1*	8
Sharma A Sharma et al. (2015)/2015	India	South Asia	Indian	CC	HB	NA	NA	*GSTM1, T1*	7
Satinder K Satinder et al. (2017)/2017	India	South Asia	Indian	CC	HB	Age	Age	*GSTM1, T1*	15
Wang J Wang et al. (2018)/2018	China	East Asia	Asian	CC	HB	NA	NA	*GSTM1*	9
Tacca A.L.M Tacca et al. (2018)/2019	Brazil	South America	Mixed	CC	HB	Age	NA	*GSTM1, T1*	13
Zhang Y Zhang et al. (2019)/2019	China	East Asia	Asian	CC	PB	NA	NA	*GSTM1*	12
Wongpratate M Wongpratate et al. (2020)/2020	Thailand	Southeast Asia	Asian	CC	PB	Age	Age	*GSTM1,T1*	14
Ueda M Ueda et al. (2008)/2008	Japan	East Asia	Asian	CC/OC	PB	NA	NA	*GSTM1, T1*	8
Sarhanis P Sarhanis et al. (1996)/1996	United Kingdom	Europe	Caucasian	OC	HB	NA	NA	*GSTM1, T1*	9
Hengstler JG Hengstler et al. (1998)/1998	Germany	Europe	Caucasian	OC	HB	NA	NA	*GSTM1,T1*	9
Goodman JE Goodman et al. (2000) 2000	Germany	Europe	Caucasian	OC	HB	NA	Age	*GSTM1, T1*	16
Lallas TA Esteller et al. (1997)/2000	United States	North America	Caucasian	OC	PB	NA	NA	*GSTM1*	10
Spurdle AB Spurdle et al. (2001)/2001	Australia	Europe	Caucasian	OC	HB	Age	Age	*GSTM1, T1*	12
Baxter SW Baxter et al. (2001)/2001	United Kingdom	Europe	Caucasian	OC	PB	NA	NA	*GSTM1*	12
Morari EC Morari et al. (2006)/2006	Brazil	South America	Mixed	OC	PB	NA	Age	*GSTM1, T1*	18
Gates M A Gates et al. (2008)/2008	United States	North America	Caucasian	OC	PB	Age	Age	*GSTM1, T1*	12
Chunhua Z Chunhua, (2008)/2008	China	East Asia	Asian	OC	BD	Age	Age	*GSTM1, T1*	11
Oliveira C Oliveira et al. (2012)/2012	Brazil	South America	Caucasian	OC	HB	NA	Age	*GSTM1, T1*	11
Khokhrin DV Khokhrin et al. (2012)/2012	Russia	Europe	Caucasian	OC	PB	NA	NA	*GSTM1, T1*	12
Cai Q Cai et al. (2016)/2016	China	East Asia	Asian	OC	PB	NA	NA	*GSTM1*	9
Pljesa I Pljesa et al. (2017)/2017	Serbia	Europe	Serbian	OC	HB	NA	Age	*GSTM1, T1*	9

SNP, single nucleotide polymorphism; OC, ovarian cancer; CC, cervical cancer.

**TABLE 2:** Basic characteristics of *GSTM1* gene polymorphism.

First author/year	Geographic region	Ethnicity	Tumor classification	Sample size	Genotypes distribution of *GSTM1* genotype			
					Cases		Controls	
					Positive	Null	Positive	Null
Warwick AP Wang et al. (2018)/1994	Europe	Caucasian	CC	77/190	37	40	96	94
Chen C Zhang et al. (2019)/1999	North America	Caucasian	CC	190/206	89	101	88	118
Kim JW Wongpratate et al. (2020)/2000	East Asia	Asian	CC	181/181	86	95	85	96
S-T CH Ueda et al. (2008)/2003	North America	Caucasian	CC	69/72	34	35	43	29
Sharma A Economopoulos et al. (2010a)/2004	South Asia	Indian	CC	142/96	61	81	63	33
Lee SA Sui et al. (2011)/2004	East Asia	Asian	CC	81/86	39	42	44	42
Niwa Y Gao et al. (2011)/2005	East Asia	Asian	CC	131/320	61	70	136	184
Sobti RC Wang et al. (2011)/2006	South Asia	Indian	CC	103/103	61	42	65	38
Zhou Q Liu and Xu, (2012)/2006	East Asia	Asian	CC	125/125	52	73	71	54
Huang YK Zhang et al. (2012)/2006	East Asia	Asian	CC	47/78	17	30	46	32
Joseph T Sun and Song, (2016)/2006	South Asia	Indian	CC	147/165	68	79	111	54
Song GY Tian et al. (2019)/2008	East Asia	Asian	CC	130/130	53	77	73	57
Singh H Zhen et al. (2013)/2008	South Asia	Indian	CC	150/168	86	64	122	46
Nishino K Sarhanis et al. (1996)/2008	East Asia	Asian	CC	124/125	47	77	66	59
De C CR Hengstler et al. (1998)/2008	South America	Mixed	CC	43/86	15	28	37	49
S-I W Goodman et al. (2000)/2009	Southeast Asia	Asian	CC	90/94	36	54	38	56
Liu Y Esteller et al. (1997)/2009	East Asia	Asian	CC	21/45	14	29	30	15
Kiran B Spurdle et al. (2001)/2010	West Asia	Caucasian	CC	46/52	21	25	22	30
Palma S Baxter et al. (2001)/2010	Europe	Caucasian	CC	25/111	10	15	53	58
Ueda M Morari et al. (2006)/2010	East Asia	Asian	CC	83/158	42	41	86	72
Stosic I Gates et al. (2008)/2014	Europa	Serbian	CC	32/50	10	22	22	28
Hasan S Chunhua, (2008)/2015	South Asia	Caucasian	CC	50/50	13	37	33	17
Natphopsuk S Oliveira et al. (2012)/2015	Southeast Asia	Asian	CC	198/198	68	130	73	125
Sharma A Khokhrin et al. (2012)/2015	South Asia	Indian	CC	135/457	56	79	297	160
Satinder K Cai et al. (2016)/2017	South Asia	Indian	CC	150/150	87	63	98	52
Wang J Pljesa et al. (2017)/2018	East Asia	Asian	CC	116/116	47	69	78	38
Tacca A.L.M Economopoulos et al. (2010b)/2019	South America	Mixed	CC	135/100	105	30	55	45
Wongpratate M Yin et al. (2013)/2020	Southeast Asia	Asian	CC	198/198	68	130	73	125
Zhang Y Jin and Hao, (2014)/2019	East Asia	Asian	CC	184/203	78	106	103	100
Ueda M Xu et al. (2014)/2008	East Asia	Asian	CC/OC	259/95	129	130	56	39
Sarhanis P Han et al. (2014)/1996	Europe	Caucasian	OC	84/312	37	47	120	192
Hengstler JG Capoluongo et al. (2006)/1998	Europe	Caucasian	OC	103/115	56	47	81	44
Lallas TA Aerssens et al. (2000)/2000	North America	Caucasian	OC	138/77	68	70	32	45
Baxter SW Moher et al. (2009)/2001	Europe	Caucasian	OC	108/106	56	47	59	40
Goodman JE Thakkinstian et al. (2011) 2000	Europe	Caucasian	OC	293/219	120	173	112	107
Spurdle AB Mantel and Haenszel, (1959)/2001	Europe	Caucasian	OC	285/299	126	159	135	162
Morari EC Der Simonian and Laird, (2015)/2006	South America	Mixed	OC	69/222	31	38	122	100
Gates M A Begg and Mazumdar, (1994)/2008	North America	Caucasian	OC	1175/1202	573	594	567	628
Chunhua Z Egger et al. (1997)/2008	East Asia	Asian	OC	89/49	58	31	43	6
Khokhrin DV Dual and Tweedie, (2000)/2012	Europe	Caucasian	OC	104/298	57	47	164	134
Oliveira C Wacholder et al. (2004)/2012	South America	Caucasian	OC	132/132	84	48	90	42
Pljesa I Ioannidis et al. (2008)/2017	Europa	Serbian	OC	85/178	44	41	89	89
Cai Q Theodoratou et al. (2012)/2016	East Asia	Asian	OC	124/124	64	60	71	53

SNP, single nucleotide polymorphism; OC, ovarian cancer; CC, cervical cancer.

**TABLE 3:** Basic characteristics of *GSTT1* gene polymorphism.

First author/year	Geographic region	Ethnicity	Tumor classification	Sample size (case/control)	Genotypes distribution of *GSTT1* genotype			
					Cases		Controls	
					Positive	Null	Positive	Null
Warwick A Tacca et al. (2018)/1994	Europe	Caucasian	CC	70/167	61	9	141	27
Chen C Zhang et al. (2019)/1999	East Asia	Asian	CC	181/181	61	120	89	92
Kim JW Wongpratate et al. (2020)/2000	South Asia	Indian	CC	142/96	114	28	84	12
Sharma A Economopoulos et al. (2010a)/2004	East Asia	Asian	CC	81/86	43	38	32	54
Lee SA Sui et al. (2011)/2004	East Asia	Asian	CC	131/320	68	63	175	145
Niwa Y Gao et al. (2011)/2005	South Asia	Indian	CC	103/103	87	16	77	26
Sobti RC Wang et al. (2011)/2006	East Asia	Asian	CC	125/125	58	67	70	55
Zhou Q Liu and Xu, (2012)/2006	South Asia	Indian	CC	147/165	123	24	149	16
Joseph T Sun and Song, (2016)/2006	South Asia	Indian	CC	150/168	110	40	150	18
Singh H Zhen et al. (2013)/2008	East Asia	Asian	CC	124/125	68	56	67	58
Nishino K Sarhanis et al. (1996)/2008	South America	Mixed	CC	43/86	21	22	70	16
De C CR Hengstler et al. (1998)/2008	Southeast Asia	Asian	CC	90/94	48	42	56	38
S-I W Goodman et al. (2000)/2009	West Asia	Caucasian	CC	46/52	31	15	36	16
Kiran B Spurdle et al. (2001)/2010	Europe	Caucasian	CC	25/111	17	8	89	22
Palma S Baxter et al. (2001)/2010	East Asia	Asian	CC	83/158	25	58	78	80
Ueda M Morari et al. (2006)/2010	Europa	Serbian	CC	32/50	20	12	30	20
Stosic I Gates et al. (2008)/2014	South Asia	Caucasian	CC	50/50	36	14	32	18
Hasan S Chunhua, (2008)/2015	South Asia	Indian	CC	135/457	109	26	392	65
Sharma A Khokhrin et al. (2012)/2015	South Asia	Indian	CC	150/150	128	22	113	37
Satinder K Cai et al. (2016)/2017	South America	Mixed	CC	135/100	69	66	44	56
Tacca A. Economopoulos et al. (2010b)/2019	Southeast Asia	Asian	CC	198/198	134	64	137	71
Wongpratate M Yin et al. (2013)/2020	East Asia	Asian	CC/OC	259/95	108	151	44	51
Ueda M Xu et al. (2014)/2008	Europe	Caucasian	OC	84/312	68	13	264	61
Sarhanis P Han et al. (2014)/1996	Europe	Caucasian	OC	103/115	87	16	99	16
Hengstler JG Capoluongo et al. (2006)/1998	Europe	Caucasian	OC	108/106	87	16	87	12
Goodman JE Thakkinstian et al. (2011) 2000	Europe	Caucasian	OC	285/299	228	57	239	56
Spurdle AB Mantel and Haenszel, (1959)/2001	South America	Mixed	OC	69/222	26	129	45	123
Morari EC Der Simonian and Laird, (2015)/2006	North America	Caucasian	OC	1175/1202	919	247	938	257
Gates M A Begg and Mazumdar, (1994)/2008	East Asia	Asian	OC	89/49	28	42	153	222
Chunhua Z Egger et al. (1997)/2008	Europe	Caucasian	OC	104/298	86	18	254	44
Khokhrin DV Dual and Tweedie, (2000)/2012	South America	Caucasian	OC	132/132	93	39	98	34
Oliveira C Wacholder et al. (2004)/2012	Europe	Serbian	OC	85/178	72	13	131	47

OC, ovarian cancer; CC, cervical cancer.

### Quantitative synthesis

#### Association of *GSTM1* present/null with the risk of CC development

**FIGURE 2:**
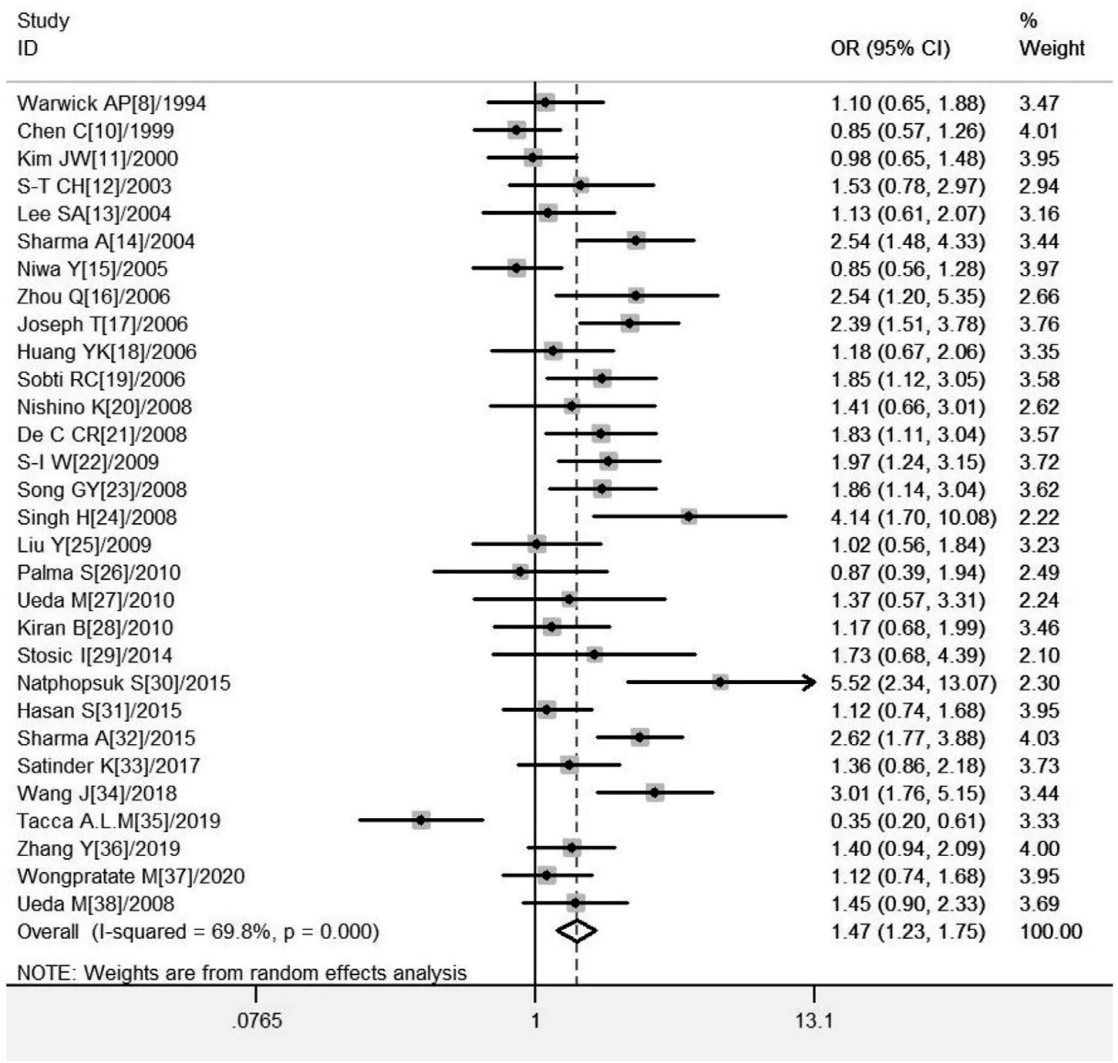
Forest plot of meta-analysis of the relationship between *GSTM1* gene polymorphisms and cervical cancer risk.

A total of 30 studies on *GSTM1* present/null polymorphisms and the risk of CC were included. Regarding the comparison of the distribution of positive vs. null in the case group and the control group, the heterogeneity test results showed that the Q test *p* = .000 and *I* ^*2*^ = 69.8%, the random effect model is used, and the forest diagram: OR [95% CI] is 1.47 (1.23–1.75), see Figure 2 and Table 4 shows the results of the association between *GSTM1* present/null polymorphisms and CC risk. In the overall analysis, individuals with *GSTM1* null genotype had a significantly increased risk of CC (OR = 1.47, 95% CI:1.23–1.75). Further subgroup analysis for race, country and geographical region showed that a significantly increased risk of CC was observed in Indians (OR = 1.96, 95% CI*:*1.51–2.55) and Asians (OR = 1.44, 95% CI:1.18–1.75), a significantly increased risk of CC was observed in East Asia (OR = 1.56, 95% CI:1.23–2.00) and South Asia (OR = 2.12, 95% CI*:* 1.58–2.85), a subgroup analysis of Asian countries showed that a significantly increased risk of CC was observed only in the Chinese population (OR = 2.10, 95% CI*:* 1.56–2.82).

**TABLE 4:** Pooled estimates of the association of GSTM1 polymorphism with risk of cervical cancer.

	n	Cases/controls	Test of association	Test of heterogeneity		Egger’s test
			OR (95% CI)	Ph	I^2^ (%)	*P*_*E*_
Overall	30	3484/4,208	1.47 (1.23–1.75)*	.000	69.8	.233
Ethnicity						
Indian	6	827/1139	1.96 (1.51–2.55)	.104	45.2	
Asian	15	1990/2152	**1.44** (1**.18–1.75**)*	.003	56.9	
Caucasian	6	457/681	**1.37** (.**85–2.21**)*	.006	69.4	
Mixed	2	178/186	**.69** (**.18–2.69**)*	.004	88.0	
Geographic region						
East Asia	12	1504/1662	1.56 (1.23–2.00)*	.002	61.8	
Europe	3	134/351	1.26 (.84–1.90)	.699	0.0	
South Asia	7	877/1189	2.12 (1.58–2.85)*	.027	57.9	
North America	2	259/278	1.07 (.61–1.88)*	.136	54.9	
Southeast Asia	3	486/490	1.10 (.85–1.42)	.963	0.0	
South America	2	178/186	.69 (.18–2.69)*	.004	88.0	
Country						
China	6	645/697	2.10 (1.56–2.82)	.134	40.6	
Japan	4	597/698	1.25 (.89–1.75)*	.109	50.5	
Korea	2	262/267	1.02 (.73–1.44)	.703	.0	
Thailand	3	486/490	1.10 (.85–1.42)	.963	.0	

*A random-effect model was used when *p* < .10 and/or *I* ^*2*^ > 50%; otherwise, a fixed-effects model was used.

Bold values means the statistical significance.

#### Association of *GSTT1* present/null with the risk of CC development

**FIGURE 3:**
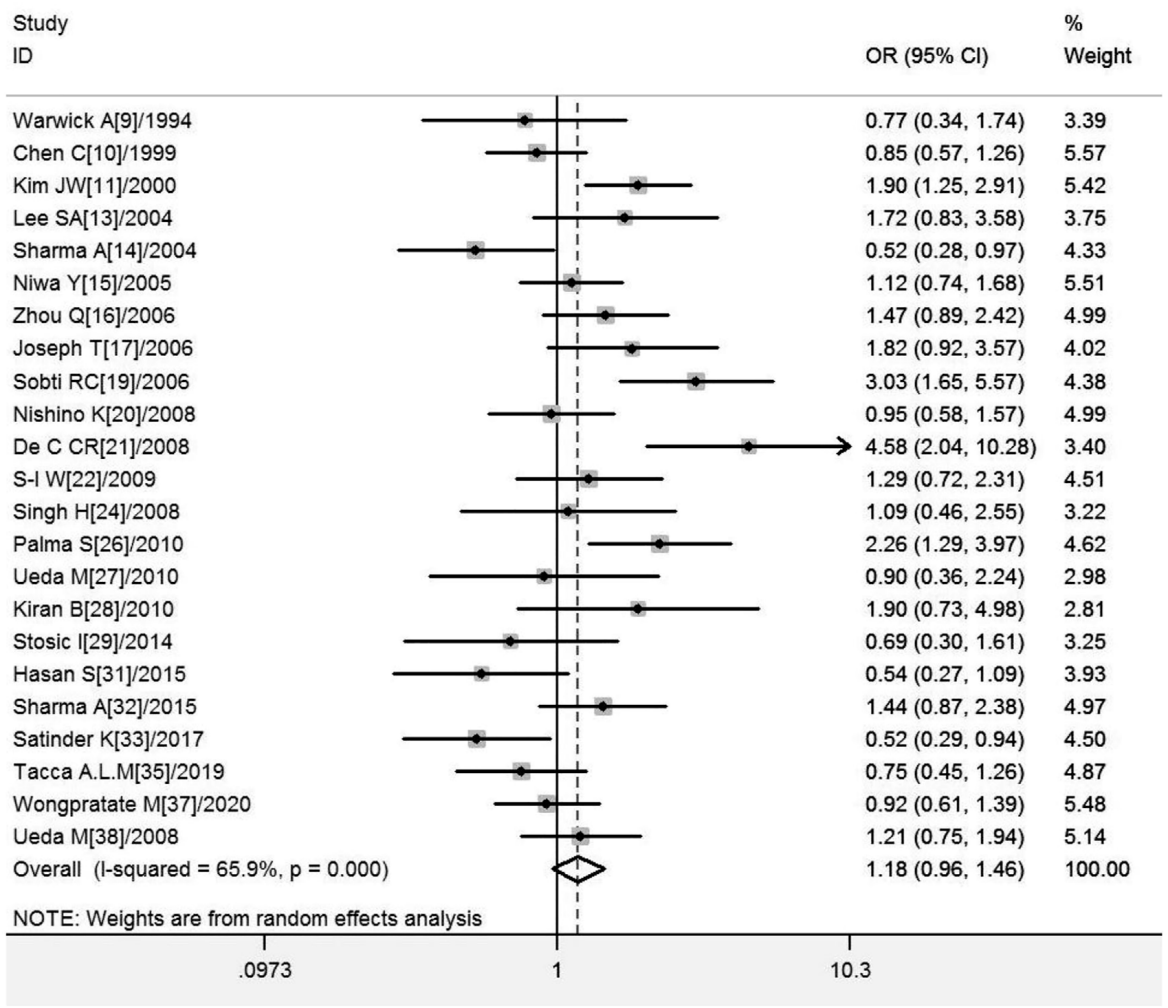
Forest plot of meta-analysis of the relationship between *GSTT1* gene polymorphisms and cervical cancer risk.

A total of 22 studies on *GSTT1* present/null polymorphisms and risk of CC were included, and the heterogeneity test showed Q-test *p* = .000 and *I* ^*2*^ = 66.0%, and the random-effects model was selected, and the forest plot showed that the OR [95% CI] was 1.21 (.97–1.50), as shown in Figure 3. Table 5 shows the results of the association between *GSTT1* present/null polymorphisms and CC risk. In the overall analysis, no association was observed between *GSTT1* null genotype and CC risk, and no association with CC risk was observed in further subgroup analysis.

**TABLE 5:** Pooled estimates of the association of GSTT1 polymorphism with risk of cervical cancer.

	n	Cases/controls	Test of association	Test of heterogeneity		Egger’s test
			OR (95% CI)	Ph	I^2^ (%)	*P*_*E*_
Overall	22	2500/3148	1.21 (.97–1.50)*	.000	66.0	.937
Ethnicity						
Indian	6	827/1139	1.25 (.72–2.20)*	.000	79.4	
Asian	9	1272/1392	1.21 (.94–1.56)*	.012	59.0	
Caucasian	4	191/381	.98 (.64–1.51)	.409	.0	
Mixed	2	178/186	1.81 (.31–10.61)*	.000	92.7	
Geographic region						
East Asia	7	984/1090	1.25 (.91–1.72)*	.008	65.6	
South Asia	7	877/1189	1.17 (.70–1.94)*	.000	77.0	
Southeast Asia	2	288/302	1.03 (.74–1.45)	.358	.0	
South America	2	178/186	1.81 (.31–10.61)*	.000	92.7	
Europe	3	127/329	1.05 (.62–1.79)	.342	6.7	

*A random-effect model was used when *p* < .10 and/or *I* ^*2*^ > 50%; otherwise, a fixed-effects model was used.

Bold values means the statistical significance.

#### Association of *GSTM1* present/null with the risk of OC development

**FIGURE 4:**
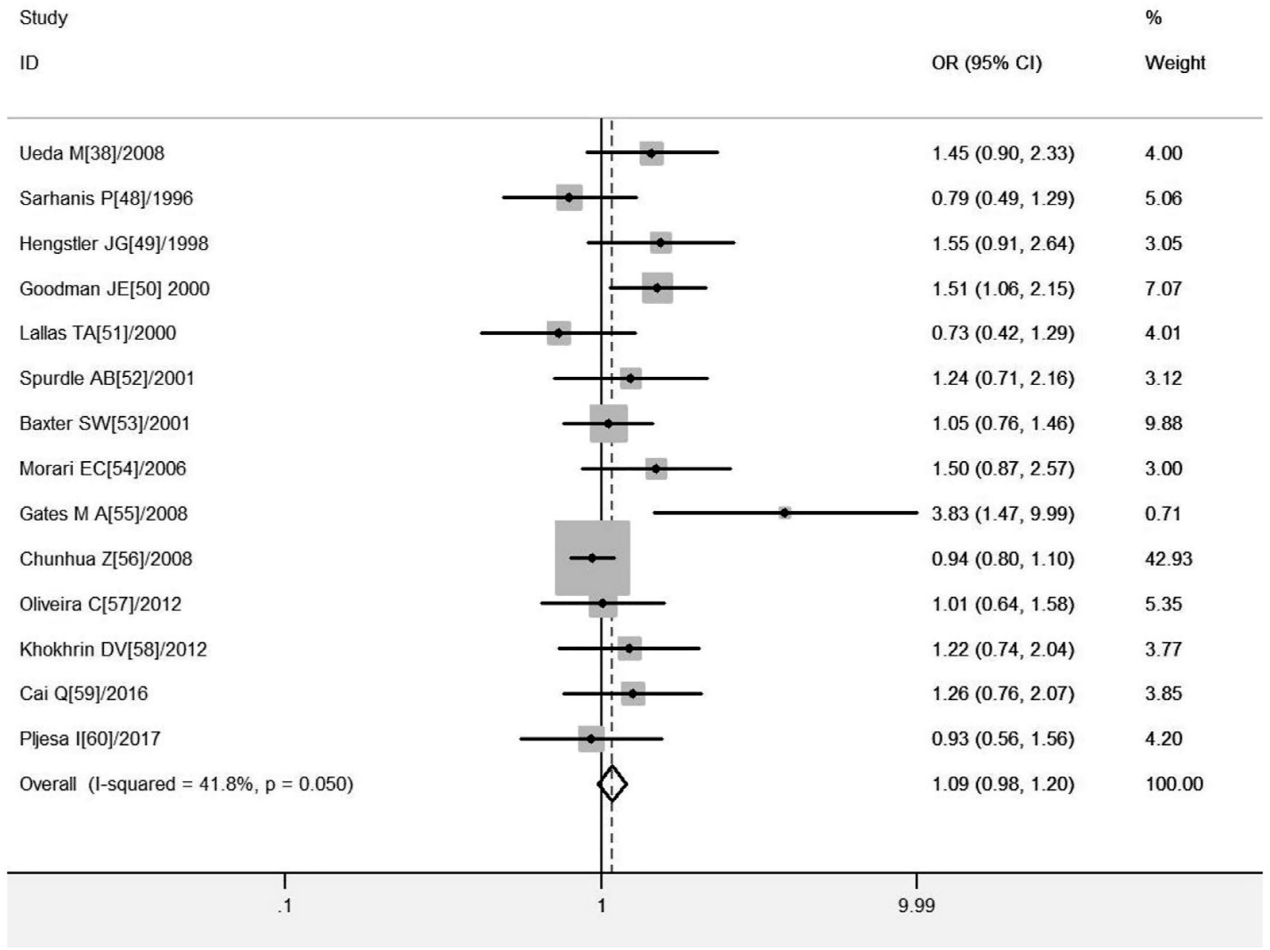
Forest plot of meta-analysis of the relationship between *GSTM1* gene polymorphisms and ovarian cancer risk.

A total of 14 studies on *GSTM1* present/null polymorphisms and risk of OC were included. The heterogeneity test showed Q-test *p* = .050 and *I* ^*2*^ = 41.8%, and a fixed-effects model was selected, and the forest plot showed that the OR [95% CI] was 1.15 (.99–1.34), as shown in Figure 4 and Table 6 shows the results of the association between *GSTM1* present/null polymorphisms and OC risk. In the overall analysis, *GSTM1* null was not significantly associated with increased OC risk, and further subgroup analysis showed that *GSTM1* null genotype was associated with increased OC risk in East Asia (OR = 1.65, 95% CI:1.00–2.73).

**TABLE 6:** Pooled estimates of the association of *GSTM1* polymorphism with risk of ovarian cancer.

	n	Cases/controls	Test of association	Test of heterogeneity		Egger’s test
			OR (95% CI)	P_*h*_	I^2^ (%)	*P*_*E*_
Overall	14	3035/3422	1.15 (.99–1.34)	.050	41.8	.044
Ethnicity						
Asian	3	472/268	1.65(1.00–2.73)*	.123	52.2	
Caucasian	9	2409/2754	1.07 (.91–1.25)	.177	30.2	
Geographic region						
East Asia	3	472/268	1.65(1.00–2.73)*	.123	52.2	
Europe	7	1057/1528	1.14 (.95–1.36)	.323	14.0	
North America	2	1305/1272	.92 (.79–1.07)	.411	.0	
South America	2	201/354	1.35 (.93–1.95)	.599	.0	

*A random-effect model was used when *p* < .10 and/or *I* ^*2*^ > 50%; otherwise, a fixed-effects model was used.

Bold values means the statistical significance.

#### Association of *GSTT1* present/null with the risk of OC development

**FIGURE 5:**
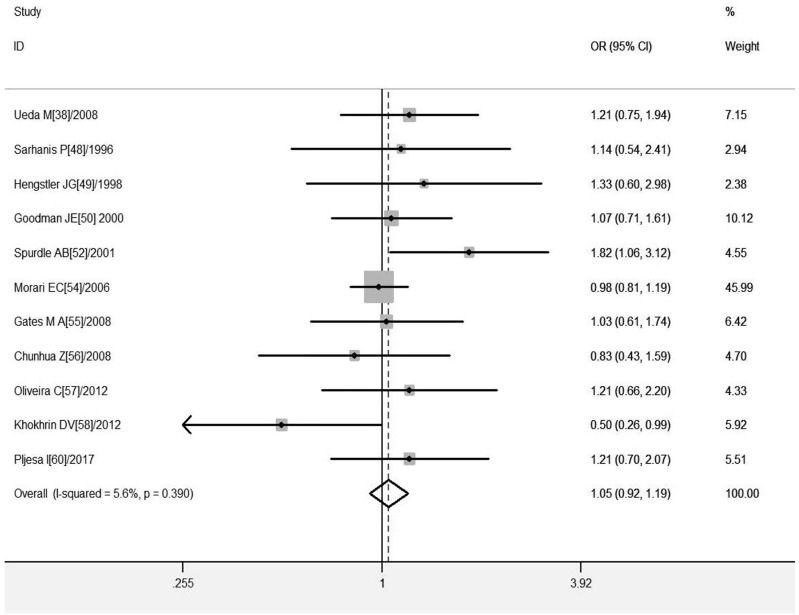
Forest plot of meta-analysis of the relationship between *GSTT1* gene polymorphisms and ovarian cancer risk.

A total of 11 studies were included regarding the *GSTT1* present/null polymorphisms and the risk of OC, and the results of the heterogeneity test showed Q-test *p* = .039 and *I* ^*2*^ = 5.6%, and the fixed-effects model was chosen, and the forest plot showed that the OR [95% CI] was 1.05 (.92–1.19), as shown in Figure 5 and Table 7 shows the results of the association between *GSTT1* present/null polymorphisms and OC risk. In the overall analysis, The *GSTT1* null genotype was not significantly associated with OC risk, but subgroup analysis showed that the *GSTT1* null genotype was associated with an increased risk of OC in South America (OR = 1.48, 95% CI:1.01–2.17).

**TABLE 7:** Pooled estimates of the association of GSTT1 polymorphism with risk of ovarian cancer.

	n	Cases/controls	Test of association	Test of heterogeneity		Egger’s test
			OR (95% CI)	Ph	I^2^ (%)	*P*_*E*_
Overall	11	2543/3275	1.05 (.92–1.19)	.039	5.6	.615
Ethnicity						
Asian	2	340/420	1.13 (.79–1.60)	.667	.0	
Caucasian	7	1986/2545	1.03 (.89–1.20)	.940	.0	
Geographic region						
East Asia	2	329/470	1.13 (.79–1.60)*	.667	.0	
Europe	6	761/1310	.97 (.76–1.24)	.378	.0	
South America	2	287/300	1.48 (1.01–2.17)	.298	7.7	

*A random-effect model was used when *p* < .10 and/or *I* ^*2*^ > 50%; otherwise, a fixed-effects model was used.

### Heterogeneity test

Due to the sources of potential heterogeneity in the individual original studies, we applied meta-regression analysis to test for heterogeneity, as shown in Table 8. In the study of *GSTM1* present/null polymorphisms and CC risk, there was heterogeneity in control matching and literature quality (*p* < .05), where matching explained 27.93% of the sources of heterogeneity and literature quality explained 18.96% of the sources of heterogeneity (not specifically reported), considering that the two types of covariates may be the main source of heterogeneity in the relevant studies. In the study of *GSTM1* present/null polymorphisms and OC risk, there was heterogeneity in sample size (*p* < .05), showing that it could explain 31.75% of the sources of heterogeneity (not specifically reported), considering that sample size could be the main source of heterogeneity in the relevant studies. No covariates were identified as a source of heterogeneity in studies of *GSTT1* present/null and risk of CC or OC.

**TABLE 8:** A) Meta-regression analysis of *GSTM1*, *GSTT1* gene polymorphisms, and risk of cervical cancer. (B) Meta-regression analysis of *GSTM1*, *GSTT1* gene polymorphisms, and risk of ovarian cancer.

(A) GSTM1		GSTT1
Logor	*P* >|t| [95% Conf. interval]	
year	.78 (−.04 to −.06)	.34 (−.11 to −.04)
Sample size	.37 (−.64 to −.24)	.94 (−.55 to −.60)
matching	.01 (−.85 to −.14)	.71 (−.61 to −.43)
adjustments	.21 (−.10 to −.45)	.83 (−.44 to −.36)
Quality score	.03 (.04 to −.79)	.51 (−.68 to −.35)
Geographic region	.71 (−.11 to −.08)	.78 (−.18 to −.14)
ethnicity	.81 (−.20 to −.16)	.81 (−.19 to −.24)
Source of controls	.07 (−.71 to −.03)	.68 (−.44 to −.66)

(B) GSTM1		GSTT1
Logor	P >|t| [95% Conf. interval]	
year	.87 (−.08 to −.09)	.49 (−.05 to −.02)
Sample size	.03 (−2.35 to −.13)	.59 (−.76 to −.46)
matching	.51 (−.48 to −.25)	.57 (−.58 to −.34)
adjustments	.71 (−.36 to −.25)	.73 (−.35 to −.48)
Quality score	.80 (−.44 to −.35)	.46 (−.29 to −.59)
Geographic region	.32 (−.29 to −.10)	.44 (−.12 to −.26)
ethnicity	.31 (−.39 to −.13)	.24 (−.41 to −.12)
Source of controls	.90 (−.39 to −.35)	.72 (−.50 to −.36)

### Sensitivity analysis

Sensitivity analysis was performed using two methods for meta-analysis. First, in evaluating the stability of the current meta-analysis, the results of each study were not changed after deleting them. Second, considering that studies with low quality and small sample size may be more likely to have positive results, we performed sensitivity analysis after excluding low-quality and small sample studies, and the results showed that *GSTM1* null was not associated with CC risk in the overall study (OR = 1.24, 95% CI:0.99–1.57), *GSTT1* null genotype was associated with CC risk in East Asia (OR = 1.45, 95% CI:1.07–1.96), *GSTM1* null genotype was not significantly associated with OC risk in East Asia, and the remaining results were not significantly changed (as shown in Tables 9).

**TABLE 9:** Pooled estimates of the association of *GSTM1*, *GSTT1* polymorphism with risk of cervical cancer or ovarian cancer. Exclude low-quality and small sample-studies.

		Cases/controls	Test of association	Test of heterogeneity	
			OR (95% CI)	Ph	I^2^ (%)
*GSTM1*with risk of cervical cancer	Overall	2126/2427	1.24 (.99–1.57)^*^	.000	72.6
	Ethnicity				
	Indian	447/483	**1.86 (1.35–2.57)**	.236	30.7
	Asian	1354/1638	**1.27 (1.05–1.53)**	.119	37.5
	Geographic region				
	East Asia	958/1242	**1.33 (1.04–1.70)** ^*^	.062	50.0
	South Asia	447/483	**1.86 (1.35–2.57)**	.236	30.7
	Southeast Asia	396/396	1.12 (.84–1.49)	1.000	.0
	Country				
	China	439/458	**1.64(1.26–2.14)**	.588	.0
	Japan	338/603	**1.16(.88–1.52)** ^*^	.067	63.0
*GSTT1* with risk of cervical cancer	Overall	1424/1700	**1.28(.94–1.75)** ^*^	.000	73.1
	Ethnicity				
	Indian	447/483	**1.42(.49–4.11)** ^*^	.000	88.6
	Asian	842/1117	**1.33(1.00–1.78)** ^*^	.039	57.4
	Geographic region				
	East Asia	644/909	**1.45(1.07–1.96)** ^*^	.082	51.6
	South Asia	447/483	**1.42(.49–4.11)** ^*^	.000	88.6
*GSTM1*with risk of ovarian cancer	Overall	2238/2640	1.05 (.94–1.18)	.286	18.2
	Ethnicity				
	Caucasian	2084/2240	1.04 (.92–1.18)	.243	25.4
	Geographic region				
	Europe	870/1091	1.16 (.96–1.39)	.464	.0
	South America	201/354	1.35 (.93–1.95)	.599	.0
*GSTT1*with risk of ovarian cancer	Overall	2030/2356	1.04 (.90–1.21)	.138	38.2
	Ethnicity				
	Caucasian	1790/2019	1.04 (.89–1.22)	.869	.0
	Geographic region				
	Europe	577/870	.98 (.74–1.29)	.179	38.9
	South America	287/300	**1.48(1.01–2.17)**	.298	7.7

* A random-effect model was used when P < 0.10 and/or I^2^ > 50%.

Bold balues means the statistical significance.

### Publication bias

**FIGURE 6:**
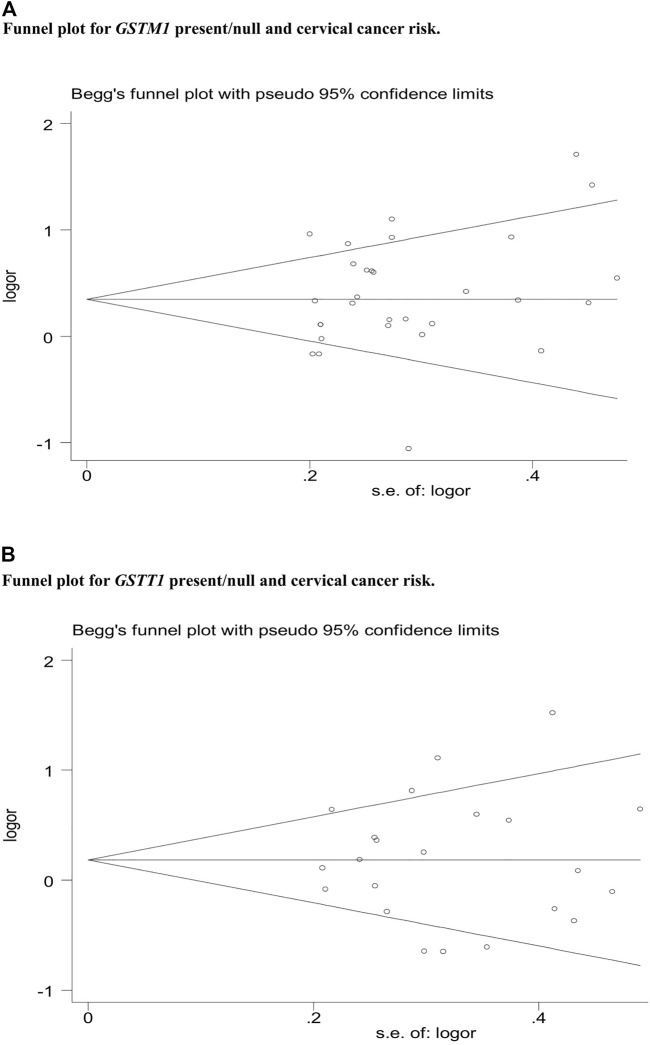
**(A)** Funnel plot for *GSTM1* present/null and cervical cancer risk. **(B)** Funnel plot for GSTT1 present/null and cervical cancer risk.

**FIGURE 7:**
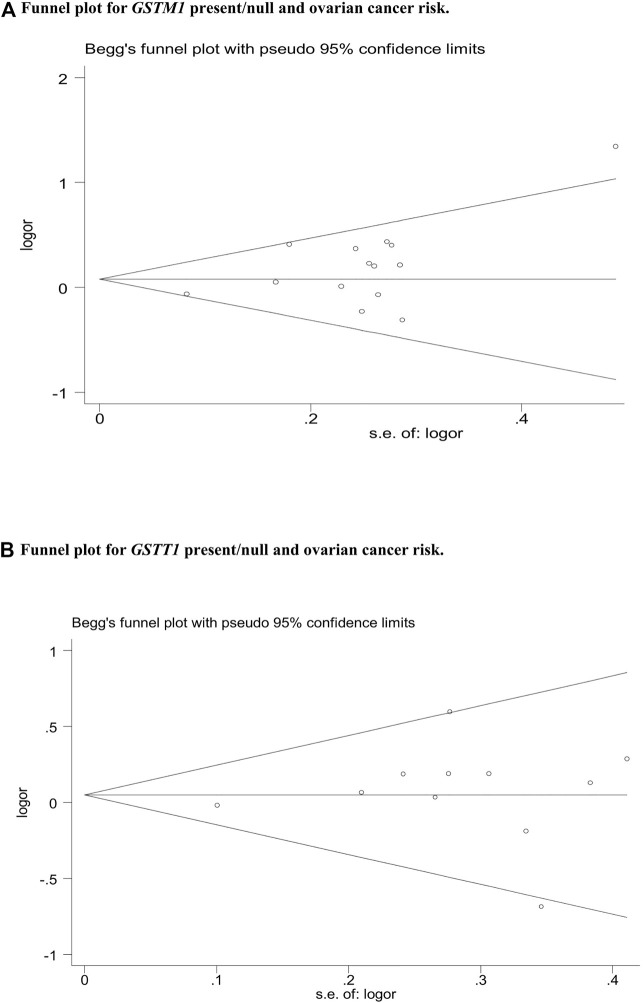
**(A)** Funnel plot for *GSTM1* present/null and ovarian cancer risk. **(B)** Funnel plot for GSTT1 present/null and ovarian cancer risk.

**FIGURE 8:**
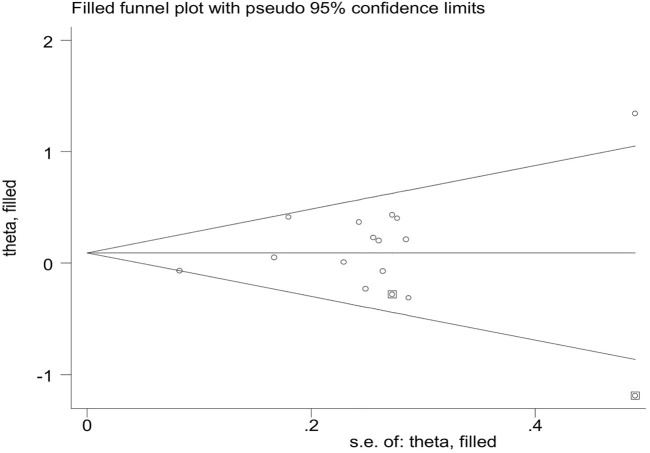
Publication bias assessed by funnel plot of *GSTM1* present/null and ovarian cancer risk.

Publication bias was assessed by Begg’s funnel plot and Egger’s test, which showed no evidence of publication bias in the studies of both the *GSTM1* present/null and *GSTT1* present/null, with the CC risk (see Figure 6). No data showed publication bias between *GSTT1* present/null polymorphisms and OC risk (see Figure 7B). The data analysis showed a bias between *GSTM1* present/null polymorphisms and OC risk (*p* = .044), as shown in Figure 7A. Further adjusted for publication bias using a non-parametric “trim and fill” approach, the results remained the same (as shown in Figure 8), indicating that the addition of studies does not affect the overall combined results.

### Reliability of positive results of current and previous meta-analyses

FPRP and BFDP can assess the likelihood of a genuine association between genetic associations and disease. We, therefore, used FPRP and BFDP to validate the credibility of the current and previous meta-analyses. An excel spreadsheet was applied to calculate FPRP and BFDP. critical values of .2 and .8 for FPRP and BRDP, respectively, were used to assess whether they were significantly associated. We determined that significant associations meeting the following statistical criteria were classified as “positive results” (Theodoratou et al., 2012): 1) *p* < .05 was observed in at least one of the two genetic models (individual the *GSTM1* present/null and *GSTT1* present/null polymorphisms, with the risk of CC or OC did not need to meet this condition, as they were only used null vs. present). 2) FPRP < .2 and BFDP < .8 at a *p*-value level of .05. 3) statistical efficacy > .8 and 4) *I* ^*2*^ < 50%. If the above criteria were not met, the association was considered a “positive result with low confidence”. Tables 10, 11, present the statistical significance associations, *I* ^*2*^ values, statistical efficacy, and FPRP and BFDP values for the current and previous meta-analyses, respectively. Based on these criteria, the results show that the positive results in the current study and the positive results of the previous meta-analysis showed “low confidence” (FPRP > .2 and BFDP > .8).

**TABLE 10:** (A) Cervical cancer false-positive report probability values for the current meta-analysis. (B) Ovarian cancer false-positive report probability values for the current meta-analysis.

(A) Variables	OR (95% CI)	I^2^ (%)	Statistical power		The prior probability of .001	
			0R = 1.2	OR = 1.5	FPRP	BFDP
GSTM1 (null vs. present)						
Overall	1.47 (1.23–1.75)	69.8	.011	.590	.568	.404
Asian	1.44 (1.18–1.75)	56.9	.033	.659	.881	.895
Indian	1.96 (1.51–2.55)	45.2	.000	.023	.806	.029
East Asia	1.56 (1.23–2.00)	61.8	.019	.379	.959	.929
South Asia	2.12 (1.58–2.85)	57.9	.000	.011	.887	.036
China	2.10 (1.56–2.82)	40.6	.000	.013	.891	.043

(B) Variables	OR (95% CI)	I^2^ (%)	Statistical power		The prior probability of .001	
			0R = 1.2	OR = 1.5	0R = 1.2	OR = 1.5
GSTM1 (null vs. present)						
Asian	1.65 (1.00–2.73)	52.2	.108	.355	.998	.998
East Asia	1.65 (1.00–2.73)	52.2	.108	.355	.998	.998
GSTT1 (null vs. present)						
South America	1.48 (1.01–2.17)	7.7	.141	.527	.997	.998

**TABLE 11:** Confidence analysis of positive results from previously published meta-analyses.

Author	Gene	Variable	OR (95% CI)	I^2^ (%)	Statistical power		The prior probability of .001	
					0R = 1.2	OR = 1.5	FPRP	BFDP
Tian Stosic et al. (2014) 2019	*GSTT1*	Overall	1.78 (1.17–2.72)	30	.034	.214	.996	.992
Sun Ueda et al. (2010) 2016	*GSTM1*	Overall	2.31 (1.57–3.40)	4.72	.000	.014	.980	.498
		HB	2.65 (1.51–4.62)	4.00	.003	.022	.996	.953
		Chinese	1.85 (1.30–2.63)	.0	.008	.121	.987	.941
		Mainland	2.33 (1.39–3.89)	4.56	.006	.046	.995	.970
Zhen Song et al. (2008) 2013	*GSTM1*	Overall	1.56 (1.39–1.75)	67	.000	.252	.000	.000
		smokers	2.27 (1.46–3.54)	.0	.002	.034	.992	.906
		Chinese	2.51 (1.73–3.65)	38	.000	.004	.963	.087
		Indians	2.07 (1.49–2.88)	41.4	.001	.028	.963	.402
		Greece	1.82 (1.11–2.99)	—	.050	.223	.997	.996
		HPV	2.25 (1.27–3.15)	61.8	.000	.009	.949	.113
Zhang de Carvalho et al. (2008) 2012	*GSTM1*	Overall	1.50 (1.21–1.85)	—	.019	.500	.891	.839
		Chinese	2.12 (1.43–3.15)	—	.002	.043	.988	.866
		Indians	2.07 (1.49–2.88)	—	.001	.028	.963	.402
		smokers	1.85 (1.07–3.20)	—	.061	.227	.998	.997
	*GSTT1*	Brazil	4.58 (2.04–5.28)	—	.001	.003	.997	.000
Liu Nishino et al. (2008) 2012	*GSTM1*	Overall	1.54 (1.18–2.00)	—	.031	.422	.975	.968
		Chinese	1.85 (1.30–2.63)	—	.008	.121	.987	.941
		Indians	2.07 (1.49–2.88)	—	.001	.028	.963	.402
		Thailand	1.02 (1.18–2.00)	—	.682	.869	.999	.999
		smokers	1.56 (1.01–2.41)	—	.119	.430	.997	.998
Wang Sobti et al. (2006) 2011	*GSTM1*	Overall	1.32 (1.06–1.66)	58.8	.208	.863	.988	.997
		Chinese	2.01 (1.46–2.79)	32.6	.001	.040	.967	.541
		Indians	1.84 (1.37–2.48)	48.5	.003	.090	.961	.686
	*GSTT1*	Latinos	4.58 (2.04–5.28)	—	.001	.003	.997	.000
Gao Huang, (2006) 2011	*GSTM1*	Cervical cancer	1.54 (1.16–2.04)	61.2	.041	.427	.985	.983
	*GSTT1*	Cervical cancer	1.49 (1.02–2.19)	69.9	.135	.514	.997	.998
		Latinos	4.58 (2.04–5.28)	—	.001	.003	.997	.000

HB, hospital-based; HPV, human papillomavirus.

## Discussion

CC and OC, as common gynecological cancers, not only impose a heavy physical and psychological burden on women worldwide but also an economic burden on their families and society. Research on genetic susceptibility in their pathogenesis has been long-standing, glutathione transferase, as one of the phase II detoxification enzymes, can catalyze the binding of glutathione to a variety of exogenous organisms and increase the water solubility and excretion of the molecule, and this detoxification ability plays a crucial role in the detoxification of glutathione S-transferase into drugs, carcinogens and reactive oxygen species. Both *GSTM1* and *GSTT1* have null genotype, which can lead to the deletion of their expression and loss of enzymatic activity, which may impair the ability of individuals to inactivate carcinogens and increase the risk of cancer. However, the results of studies related to the risk of CC or OC by *GSTM1* and *GSTT1* are inconsistent or even contradictory, so we performed a new statistical analysis of previous and newly published studies to obtain more accurate evidence-based medical conclusion.

Overall, in the current meta-analysis, statistically significant null of the *GSTM1* increased the risk of CC, and based on the biochemical characteristics of *GSTM1* present/null polymorphisms. We estimated that individual effects of these genes were associated with an increased risk of CC in all ethnic groups. However, the risk was not consistent across populations, and studies showed that only in Indian and Chinese populations was the risk of CC significantly the increased risk was observed only in Indian and Chinese populations, and no risk correlation was observed in Caucasian and mixed populations, etc., Which may be due to the association of CC development with environmental factors. In addition, in studies related to OC risk, *GSTM1* null was shown to be associated with an increased risk of OC in East Asia. *GSTT1* null genotype was associated with an increased risk of OC in South America; while no correlation was found in other regions and populations. These results suggest, that the same genes may play different roles in cancer susceptibility across ethnicities and geographic regions. Because cancer is a complex polygenic disease and different genetic backgrounds and environmental factors (economic conditions or lifestyle) may contribute to such differences. Furthermore, random errors and biases are often found in some small-sample, low-quality studies in control groups, so the results of these original studies are not credible, especially in studies of genetic polymorphisms and disease susceptibility. In addition, small sample studies with positive results may be more likely to be reported, however, when they tend to achieve positive results, their studies may be less rigorous and often of lower quality (Attia et al., 2003). Therefore, we assessed the sensitivity analysis to see if there was any variation in the results by including only high-quality and large sample studies, and finally used FPRP and BFDP tests to assess the association between the positive findings from the current meta-analysis and the results of previous relevant studies, as FPRP is considered an appropriate method to assess the probability of significant results in multiple hypothesis testing of genetic polymorphisms and disease susceptibility studies, and In turn, Wacholder et al. (2004) provided a more precise genetic test, and the two methods together further strengthen the confidence of the conclusions, the results of the test on the current study showed that in *GSTM1* null may be associated with an increased risk of CC and *GSTM1* and *GSTT1* null may be associated with an increased risk of OC, but the associated positive results showed “low confidence” (FPRP > .2, BFDP > .8).

A total of nine previous studies have been published on the association between individual *GSTM1* and/or *GSTT1* present/null polymorphisms and CC risk (Economopoulos et al., 2010a; Gao et al., 2011; Sui et al., 2011; Wang et al., 2011; Liu and Xu, 2012; Zhang et al., 2012; Zhen et al., 2013; Sun and Song, 2016; Tian et al., 2019), Economopoulos et al. (2010a) published a meta-analysis showing that *GSTM1* null increases the risk of CC in non-Chinese, while Sui et al. (2011) showed in a published study that *GSTM1*, *GSTT1* null was not associated with CC risk, Gao et al. (2011) suggested in a published study that individual *GSTM1* and *GSTT1* null increased the risk of CC in the entire study population, in a meta-analysis published by Wang et al. (2011), Liu and Xu (2012), Zhang et al. (2012), Zheng et al. (2013) and Sun and Song (2016) all concluded that *GSTM1* null increased the risk of CC in the overall study, smokers, Indians and Chinese, but not in Koreans, while in the Japanese population or other ethnic groups, such as Caucasians, Wang et al. (2011), and Zhang et al. (2012) also performed a combined analysis of *GSTT1* null genotype and CC risk, and all results showed no significant association with CC risk. Although the results of the latest meta-analysis published by Tian et al. (2019) were not fully consistent with the previous results, the analysis of results observed that a single *GSTM1* null genotype was not associated with an increased risk of CC, whereas *GSTT1* null increased the risk of CC in the whole study. Five previous papers have summarized the association between individual *GSTM1* and/or *GSTT1* present/null polymorphisms and OC risk, concluding that none of the studies observed any association with OC risk except for the finding by Jin et al. (Xu et al., 2014) showing that *GSTT1* null increases OC risk in Asian populations. In addition, previously published studies had several shortcomings, *I* ^*2*^ values were not shown in two meta-analyses (Liu and Xu, 2012; Zhang et al., 2012). Ten meta-analyses did not assess the quality of eligible studies (Capoluongo et al., 2006; Economopoulos et al., 2010a; Gao et al., 2011; Sui et al., 2011; Wang et al., 2011; Liu and Xu, 2012; Yin et al., 2013; Han et al., 2014; Jin and Hao, 2014; Xu et al., 2014), all meta-analyses did not look for sources of heterogeneity, and the probability and statistical significance of false positive reports were not assessed (Capoluongo et al., 2006; Economopoulos et al., 2010a; Gao et al., 2011; Sui et al., 2011; Wang et al., 2011; Liu and Xu, 2012; Zhang et al., 2012; Yin et al., 2013; Zhen et al., 2013; Han et al., 2014; Jin and Hao, 2014; Xu et al., 2014; Sun and Song, 2016; Tian et al., 2019). Therefore, by assessing the degree of association between positive results, the results showed that their meta-analysis results may not be credible (all meta-analyses FPRP> .2, BFDP> .8) (as shown in Table 11).

Compared with previous meta-analyses, this meta-analysis has several advantages: First, in addition to the inclusion of newly published original studies, the sample size was larger, including 30 studies of *GSTM1* gene polymorphism (3,484 cases and 4,208 controls) and 22 studies of *GSTT1* present/null polymorphisms (2,500 cases and 3,148 controls) associated with the risk of CC, and OC risk included 14 studies of *GSTM1* present/null polymorphisms (3,035 cases and 3,422 controls) and 11 studies of *GSTT1* present/null polymorphisms (2,543 cases and 3,275 controls). Second, we performed a quality assessment of the included eligible studies. Third, we applied FPRP and BFDP tests to assess false positive associations to estimate positive findings from this meta-analysis and previous relevant studies. Fourth, meta-regression analysis was applied to explore the sources of heterogeneity. Fifth, important sensitivity analyses were performed for studies with high-quality and large samples. However, our meta-analysis has some limitations: First, some potential covariates were not controlled for, such as age. Second, in the subgroup analysis, although some population studies showed positive results, for example, in the study on the association between *GSTM1* and/or *GSTT1* present/null polymorphisms and CC risk, the results on South American countries showed that *GSTT1* null genotype reduced the risk of CC, and in studies on the association between *GSTM1* and/or *GSTT1* null genotype and OC risk, *GSTT1* null genotype was found to increase the risk of OC in mixed ethnic and Serbian populations. However, the positive results of the above studies corresponded to only one study each (not specifically reported) and the sample size was small enough to explore the true association between them and confirm the validity of their results, so a large sample size and sufficiently large studies would help to validate our findings. Third, the current meta-analysis included only published articles, so there may be publication bias, as shown in Figure 8; known positive results are more likely to be published than negative results, so the genetic effect of *GSTM1* and *GSTT1* null genotype may be underestimated. Fourth, we did not consider whether the genotype distribution in the controls was in Hardy–Weinberg equilibrium (HWE). Under normal circumstances, the HWE in the meta-analysis of genetic polymorphisms must be calculated to assess the quality, genotyping errors, and selection bias in the study (Hosking et al., 2004; Thakkinstian et al., 2011). However, we cannot calculate or extract the relevant data in the original studies. Fifth, for CC, data on other risk factors such as HPV infection, age and smoking were not extracted, while for ovarian cancer, data on age, obesity and tumor pathological classification were not extracted.

## Conclusion

The results of this meta-analysis study suggest that the positive results of *GSTM1* null genotype associated with increased risk of CC, and *GSTM1* and *GSTT1* null genotype associated with increased risk of OC in Chinese and Indian populations may be results with missing credibility rather than true associations, and therefore we should interpret these positive results with caution. In conclusion, due to the small sample size of the relevant studies and the limitations of this study, the *GSTM1* present/null and/or *GSTT1* present/null polymorphisms with risk of CC or OC still needs to be further explored in depth, and we need more original studies with larger samples for validation.

## Data Availability

The datasets presented in this study can be found in online repositories. The names of the repository/repositories and accession number(s) can be found in the article/Supplementary Material.
