# Supplementary material for: Individual effects of GSTM1 and GSTT1 polymorphisms on cervical or ovarian cancer risk: An updated meta-analysis
Source: Front Genet. 2023 Jan 12;13:1074570. doi: 10.3389/fgene.2022.1074570 (PMC9879013; doi:10.3389/fgene.2022.1074570)
Supplement: Supplementary file 1 [file DataSheet1.pdf]

Table SI. Literature quality score

Criterion	Score
Source of case	
Selected from population or cancer registry	3
Selected from hospital	2
Selected from pathology archives, but without description	1
Not described	0
Source of control	
Population-based	3
Blood donors or volunteers	2
Hospital-based	1
Not described	0
Ascertainment of cancer	
Histological or pathological confirmation	2
Diagnosis of cancer by patient medical record	1
Not described	0
Ascertainment of control	
Controls were tested to screen out CC、OC	2
Controls were subjects who did not report CC、OC, no objective testing	1
Not described	0
Matching	
Controls matched with cases only by age	2
Not matched or not described	0
Genotyping examination	
Genotyping done blindly and quality control	2
Only genotyping done blindly or quality control	1
Unblinded and without quality control	0
Specimens used for determining genotypes	
Blood cells or normal tissues	1
Tumor tissues or exfoliated cells of tissue	0
Correlation between genotype and CC, OC	
Correct statistics (control for confounding factors)	2
Correct statistics (without controlling for confounding factors)	1
Statistics with biases	0
Total sample size	
＞1000	3
500-1000	2
200-500	1
<200	0

CC: Cervical Cancer; OC: Ovarian Cancer.
